# The Great Fraud on the Doctors: Substitution

**Published:** 1901-09

**Authors:** 


### TEXAS MEDICAL JOURNAL.

AUSTIN, TEXAS.

A MONTHLY JOURNAL OF MEDICINE AND SURGERY.

EDITED AND PUBLISHED BY
F. E. DANIEL, M. D.

ASSOCIATE EDITOR:
WITTEN BOOTH RUSS, M. D.,
San Antonio, Texas.

Published Monthly at Austin, Texas. Subscription price $1.00 a year in advance.

Eastern Representative: John Guy Monihan, St. Paul Building, 220 Broadway, New York City.

Official organ of the State Association of Health Officers, the West Texas Medical Association, the Houston District Medical Association, the Austin District Medical Society, the Brazos Valley Medical Association, the Galveston County Medical Society, and several others.

### THE GREAT FRAUD ON THE DOCTORS: SUBSTITUTION.

This evil is constantly growing and has become a most serious matter. The practitioner of medicine owes it to himself, to his reputation, to his family, and, above all, to those who entrust their lives and the lives of their dear ones to his care, to take active steps to put a stop to this dangerous, swindling practice. There is but one way to do it. Let every physician *see to it* that he is not swindled and his patient imposed upon, and his life jeopardized by a money grabbing druggist without conscience; and if one of these fellows be caught, let him be denounced in the medical society; branded as unworthy of confidence; boycotted by every self-respecting physician. The average physician has not given the matter serious thought. This is how it works. You prescribe a true and tried remedy, and confidently expect certain results. You don't get them. The family are disappointed. The prescription has been intended to meet a crisis. It didn't connect. The child died. The family get another physician, and you wonder why? Your druggist has gotten in his work—put up "something just as good," and there you are. My dear readers, get a move on you. This hydra-headed monster must be put down, and it can only be done by prompt action on your part.

I extract the following pointed remarks from a letter—one of many I have received on the subject—from an esteemed subscriber, and I endorse it, and commend it to the careful, thoughtful consideration of the grand army of readers of the "Red Back":

"It has always been a matter of surprise to me that the average doctor who, under the most favorable conditions, has as many cares and burdens as will be found in any walk of life, should not endeavor to cast off some of them, and lighten his load of trouble as much as possible. After having spent many years, much money and a great deal of gray matter in equipping himself to practice medicine and is able to do so most successfully, he pays no attention to the fact that his prescriptions have not been properly filled, and not only does his patient fail to be benefited at the risk of health, or perhaps of life, but his own reputation is apt to be seriously impaired thereby. It is a notorious fact that substitution among retail druggists, and especially on prescriptions for proprietary articles, is one of the crying evils of the day. To try to correct this by exposing the guilty party, or by bringing legal action against him, is apt to be attended with much annoyance and expense and, in this way, it is almost impossible to reach the hundreds and hundreds of offenders.

"Now, if the physician could be made to understand the vital importance of directing his patients to the most reliable druggists, of prescribing in original packages or of examining the prescriptions after they are filled, the difficulty might be overcome. No retail druggist would dare to continue this nefarious practice, if he understood that, in case of detection, the physician would not only expose him to all of his patients, but would report him to his medical society, and warn his fellow practitioners against sending or giving him any patronage.

"There is no telling how much harm has been done to the reputation of many competent medical men, simply because unscrupulous druggists did not dispense the genuine article on a prescription, for the expected results did not materialize, the patient became disgusted, and naturally blamed the doctor."

In our June number I reproduced the Tennessee anti-substitution bill. It was written by that sterling old warrior-physician, Deering J. Roberts, than whom a more worthy, conscientious and ethical physician does not exist, and it was mainly by his efforts that it passed and became a law. I call attention to this bill and ask that the subject be taken up in all the medical societies. Let each society send up a petition or memorial to the next Texas Legislature, asking that a similar bill be passed. I will personally draw up the bill and use my best efforts to get it through. Meantime let every individual member see to it that he and his patient get what he orders, or know the reason why. No man would stand substitution by his grocer, for instance, but many men, doctors, seem to be indifferent to the fact that substitution of some worthless stuff in his prescriptions is being practiced daily to the danger of his reputation and practice and to his patient's life. If a proprietary medicine be ordered, see that the prescription is filled from the original bottle, seal unbroken. Make Mr. Druggist open a new bottle.

Rouse ye, Romans! Self-protection is the first law of nature. To protect your reputation and your interest, is like unto it. To protect the lives of your patrons is a duty as imperative. There is but one way to do it. Go for the thieving, unscrupulous, conscienceless substitutor in a way,—the only way you can reach him. Boycott him! Brand him, *as he deserves,* as a *felon.*

